# Two-megahertz impedance index prediction equation for appendicular lean mass in Korean older people

**DOI:** 10.1186/s12877-022-02997-6

**Published:** 2022-05-02

**Authors:** Hyeoijin Kim, Keon-Hyoung Song, Jatin P. Ambegaonkar, Sochung Chung, Kwonchan Jeon, Fang Lin Jiang, Jin Jong Eom, Chul-Hyun Kim

**Affiliations:** 1 Department of Physical Education, Korean National University of Education, Cheongju, Republic of Korea; 2 Department of Pharmaceutical Engineering, Soonchunhyang University, Asan, Republic of Korea; 3 SMART Laboratory, School of Kinesiology, George Mason University, Manassas, VA USA; 4 Department of Pediatrics, Konkuk University Medical Center, Konkuk University School of Medicine, Seoul, Republic of Korea; 5 School of Health Sciences, Public Health Program, Salisbury University, Salisbury, MD USA; 6 Department of Sports Medicine, Soonchunhyang University, Asan, Republic of Korea; 7 National Traditional Sports Teaching and Research Section of Hunan Province, College of Physical Education, Hunan Narmal University, Changsha, China; 8 Department of Sport, Leisure & Recreation, Soonchunhyang University, Asan, Republic of Korea

**Keywords:** Appendicular skeletal muscle mass, Diagnosis, Multifrequency Bioelectrical Impedance Analysis, Validation, Algorithm, Dual-energy X-ray Absorptiometry

## Abstract

**Background:**

Whole-body bioelectrical impedance analysis (BIA) has been accepted as an indirect method to estimate appendicular lean mass (ALM) comparable to dual-energy X-ray absorptiometry (DXA). However, single or limited frequencies currently used for these estimates may over or under-estimate ALM. Accordingly, there is a need to measure the impedance parameter with appendicular lean-specific across multiple frequencies to more accurately estimate ALM. We aimed to validate muscle-specific frequency BIA equation for ALM using multifrequency BIA (MF-BIA) with DXA as the reference.

**Methods:**

195 community-dwelling Korean older people (94 men and 101 women) aged 70 ~ 92y participated in this study. ALM was measured by DXA and bioimpedance measures at frequencies of 5 kHz ~ 3 MHz were assessed for independent predictive variables. Regression analyses were used to find limb-specific frequencies of bioimpedance, to develop the ALM equations and to conduct the internal cross-validation. The six published equations and the final equation of MF-BIA were externally cross-validated.

**Results:**

195 participants completed the measurements of MF-BIA and DXA. Using bivariate regression analysis, the 2 MHz impedance index explained *R*^2^ = 91.5% of variability (*P* < 0.001) in ALM and predictive accuracy of standard error of estimate (SEE) was 1.0822 kg ALM (*P* < 0.001). Multiple stepwise regression analysis obtained in the development group had an adjusted *R*^2^ of 9.28% (*P* < 0.001) and a SEE of 0.97 kg ALM. The cross-validation group had no significant difference between the measured ALM and the predicted ALM (17.8 ± 3.9 kg vs. 17.7 ± 3.8 kg, *P* = .486) with 93.1% of *R*^2^ (*P* < 0.001) and 1.00 kg ALM of total error. The final regression equation was as follows: ALM = 0.247*ZI*_@2 MHz_ + 1.254SEX_M1F0_ + 0.067*Xc*_@5 kHz_ + 1.739 with 93% of *R*^2^ (*P* < 0.001), 0.97 kg ALM of SEE (Subjective Rating as “excellent” for men and “very good” for women). In the analysis of the diagnostic level for sarcopenia of the final regression, the overall agreement was 94.9% (*k* = 0.779, *P* < 0.001) with 71.4% of sensitivity, 98.8% of specificity, 91.3 of positive prediction value and 95.3% of negative prediction value.

**Conclusion:**

The newly developed appendicular lean-specific high-frequency BIA prediction equation has a high predictive accuracy, sensitivity, specificity, and agreement for both individual and group measurements. Thus, the high-frequency BIA prediction equation is suitable not only for epidemiological studies, but also for the diagnosis of sarcopenia in clinical settings.

## Introduction

The term “sarcopenia” in 1989 was defined as the loss of skeletal muscle mass with advancing age in an elderly population [1]. The advanced definition of sarcopenia in clinical practice and research has been extended to include a decrease in muscle strength and/or physical performance. In 2016, the World Health Organization (WHO) classified sarcopenia with these conditions as a muscle disease with the diagnostic ICD-10-CM: M62.84 code [1, 2]. In 2019, based on recent scientific evidence, the European Working Group on Sarcopenia in Older People 2 (EWGSOP2) redefined sarcopenia as “a progressive and generalized skeletal muscle disorder that is associated with increased likelihood of adverse outcomes including falls, fractures, physical disability, and mortality”. EWGSOP2 updated its algorithm for sarcopenia case-finding, diagnosis, and severity determination in addition to recommending a four-step pathway of Find-Assess-Confirm-Severity (F-A-C-S) [3]. At the third step of Confirm, appendicular lean mass (ALM) from the dual-energy X-ray absorptiometry (DXA) is recommended as the values of the skeletal muscle mass to use for sarcopenia diagnose [3–7].

DXA is a sophisticated machine that produces two X-ray beams—consisting of attenuation of photons at two different energies (i.e., 40 keV and 70 keV)—passing through the human body to evaluate bone mineral content, body fat mass, soft lean tissue mass and ALM by computing every pixel of DXA images. DXA has been widely validated to assess ALM, as it is an accurate measure and the reference standard of muscle mass in four limbs (i.e., arms and legs) for the diagnosis of sarcopenia. However, the DXA has limitations for use in clinical setting or epidemiological studies due to the high cost and large size of equipment, technical expertise, and risk of radiation exposure [8, 9]. Under consideration of DXA’s limitations, the consensus expert groups for Sarcopenia have accepted the bioelectrical impedance analysis (BIA) as a doubly indirect method to estimate accurate ALM. BIA has benefits including ease of use, safety, low cost, portability, and high reliability [3, 6, 8–10].

In BIA, electrical currents pass through the human body to measure the body resistance (*R*), reactance (*X*_*C*_), and impedance (*Z*). The software system then estimates extracellular fluid (ECF), total body water (TBW) and fat-free mass (FFM) based on the impedance index (*ZI*), which is the ratio between squared height (Ht^2^) and body impedance (*Z*). *ZI* of approximately ~ 1.25 MHz can be used due to a high correlation (up to ~ 0.960) with ECF and TBW [11, 12]. By adjusting the mediating variables of ethnicity, age group, and gender, BIA may be able to give a more accurate prediction of TBW, FFM, and ASM through *ZI* that is comparable to DXA [13]. The devices and equations of the single-frequency whole-body BIA at 50 kHz have been recently developed for estimating ALM [14–22]. However, a single frequency of 50 kHz, and whole-body bioimpedance measurements (Z, *R*, and *Xc*) in present use can be improved by using tissue-specific multifrequency bioimpedance measurements [12, 23, 24].

Single frequency at 50 kHz or the limited range of multifrequency from 5 to 250 kHz that has been currently applied to estimate ALM has considerably lower conductivity and lower correlation coefficient with TBW than the higher range of multifrequency to 1.25 MHz. Consequently, these factors may lead to excessive predictive estimation error and can cause the over-estimations or under-estimations of ALM [11, 12, 23, 25]. Further, skeletal muscle tissue has improved conductivity at 1 MHz or higher, and not at 50 kHz frequency, and the capacitor, or reactance, at frequencies around 50 kHz accurately estimates the amount of skeletal muscle cells [11, 24]. It is necessary to have the impedance measures with appendicular lean-specific wide multifrequency for the more accurate estimation of ALM.

Thus, in the current study we aimed to validate the muscle-specific frequency BIA prediction equation for ALM by multifrequency (i.e., 5kz, 50 kHz, 250 kHz, 500 kHz, 1 MHz, 2 MHz and 3 MHz) BIA using DXA as the criterion reference.

## Methods

### Study population

Participants in this study comprised of a total of 195 Korean urban community-dwelling older adults aged 70 – 92 years (94 men and 101 women) who live within Seoul, South Korea, a major urban metropolis were participated in this study. All participants in this study were recruited through advertisements in local newspapers or social networks, advertisements on public welfare communities, or through notices from public welfare centers. Participants with critical illness, clinical disease (heart failure, renal failure), hospitalization within 3 months, more than 6 kg weight loss within 6 months of measurement, unable to maintain a standing position, indwelling devices and complete or partial amputation of one or more limbs were excluded from the study. These participants were instructed both fast for at least 4 h, avoiding vigorous activity 8 h before testing, and restrict drinking alcohol for 12 h prior to measurements at the Body Composition Laboratory. The participant’s weight was measured using a precision scale (Model DB-1, CAS, South Korea) in units of 0.05 kg. The height was measured in 0.1 cm increments using a SECA 274® stadiometer (Hamburg, Germany).

### Ethics and informed consent statement

The study design and procedures were reviewed by the ethics committee of the Korean National Sport University and were approved by that ethics committee, under reference No.1263201903HR0102. All the Participants were apparently healthy without physical disability and received a written informed consent. All procedures of this study have been performed in accordance with the Declaration of Helsinki.

### Measurement of ALM mass by DXA

Each participant’s total and appendicular bone mineral content, fat and lean body mass was measured through whole-body DXA scan (Prodigy Advance, GE Lunar, USA). The Spine Phantom tool provided by the manufacturer was used to calibrate the DXA equipment for daily quality control purposes. iDXA software (version 4.0.2) was used to analyze all scans from the original DXA performed by a single operator for imaging scan standardization. The regions of interest (ROIs) for ASM were assessed using the measurement published by Jeon et al. [26]. After the manual analysis of ROIs, All DXA scans give it the measurement for body composition including the whole and appendicular bone minaral content, fat and lean body mass as well as appendicular skeletal muscle mass.

*Measurement of multifrequency impedance parameters for ALM* mass.

In this study, All the measurements were performed in the supine position on a non-conductive platform using MF-BIA (BWA 2.0, InBody Co. South Korea). Eight electrodes were placed on the dorsal surfaces of the right and left hands, wrists, ankles, and feet during measurements, as previously described [26]. Briefly, voltage electrodes were placed the styloid process of the ulna and medial malleolus of the right and left, and current electrodes were placed on the metatarsophalangeal joints of the right and left hands and feet. Impedance parameters were measured using multiple frequencies (1 kHz, 5 kHz, 50 kHz, 250 kHz, 500 kHz, 1 MHz, 2 MHz, and 3 MHz) and alternating current (300µA) which is verified as safe and has no electrical burden on the human body [27, 28]. The measurements by MF-BIA also calculated values for index height^2^/resistance (i.e., *R* index, *RI*) and index height^2^/impedance (i.e., *Z* index, *ZI*). The device was calibrate using the manufacture manual for the standard control circuit when conducting the measurements. A value of less than 2% is used for the precision error of FFM in the study.

### Published prediction equations for the external cross-validation

The published single-frequency whole-body BIA-based equations and the newly developed equations in this study were used for external cross-validation and overall agreement of the diagnostic classification of sarcopenia with this study population. The prediction of ALM was estimated according to the equations previously published studies presented in Table 4 [15–20] as previously described [26].

### Statistical Analysis

Statistical analyses were performed using SPSS version 26 (IBM, USA). Descriptive statistics for the physical characteristics of participants from the development and cross-validation groups were expressed as mean ± standard deviation (SD), and ranges. Differences between mean values of gender from both development and cross-validation groups were assessed by independent *t* tests. The ALM_@xHz_ equations in the development group were developed by the multiple stepwise linear regression. The goodness-of-fit tests were conducted to determine the developmental equations and the internal and external cross-validation study [26]. In addition, Bland–Altman plots were performed to examine limits of agreement (LoA), bias, and percentage of individual agreement (PIA) within the minimally acceptable standard for prediction errors [26]. Cohen’s Kappa was conducted for the overall agreement between the diagnostic classification of sarcopenia by BIA equations and DXA measurements. Statistical significance was set at a *p* value of < 0.05.

## Results

### Characteristics of the Study Population

The total of 195 (men = 94, women = 101), who completed this study, were randomly assigned into a development group (*n* = 131) and an internal cross-validation group (*n* = 64), respectively. Table 1 shows the physical characteristics and body composition variables for each group.

**Table 1:** Characterisitics of the participants for eqation development and cross-validation

	Development Group		Cross-validation Group	
	Men (*n* = 63)	Women (*n* = 68)	Men (*n* = 31)	Women (*n* = 33)
Age (years)	77.5 ± 4.1	76.9 ± 4.4	76.5 ± 4.4	76.9 ± 3.7
Height (cm)	166 ± 5.0	153 ± 4.5^*^	168 ± 4.5	153 ± 5.0^*^
Weight (kg)	65.0 ± 7.5	55.3 ± 6.5^*^	67.2 ± 7.0	55.8 ± 5.5^*^
BMI (kg/m^2^)	23.5 ± 2.4	23.6 ± 2.5	23.7 ± 2.4	23.8 ± 2.1
FFM (kg)	49.8 ± 4.3	37.2 ± 3.4^*^	51.6 ± 4.2^†^	37.8 ± 3.5^*^
FM (kg)	15.2 ± 5.8	18.1 ± 4.7^*^	15.6 ± 4.3	17.9 ± 3.5^*^
PBF (%)	22.9 ± 6.9	32.4 ± 5.4^*^	22.8 ± 5.0	32.0 ± 4.3^*^
ALM (kg)	20.4 ± 2.2	14.3 ± 1.6^*^	21.3 ± 2.1	14.4 ± 1.4^*^
*Xc*_@5 kHz_	13.6 ± 4.1	12.5 ± 3.4	13.8 ± 3.3	14.0 ± 3.2^†^
*Xc*_@50 kHz_	38.0 ± 6.6	37.8 ± 5.7	37.4 ± 4.5	39.8 ± 5.4
*Xc*_@250 kHz_	28.6 ± 3.8	30.5 ± 3.4^*^	27.7 ± 2.5	31.6 ± 3.3^*^
*R*_@5 kHz_	563 ± 57	656 ± 66^*^	553 ± 49	657 ± 46^*^
*R*_@50 kHz_	502 ± 50	595 ± 60^*^	493 ± 46	594 ± 42^*^
*R*_@250 kHz_	453 ± 45	543 ± 55^*^	445 ± 42	539 ± 38^*^
*ZI*_@1 kHz_	48.6 ± 6.0	35.4 ± 4.1^*^	50.4 ± 5.4	35.0 ± 3.9^*^
*ZI*_@5 kHz_	49.6 ± 6.1	36.1 ± 4.1^*^	51.5 ± 5.5	35.8 ± 3.9^*^
*ZI*_@50 kHz_	55.4 ± 6.6	39.7 ± 4.6^*^	57.8 ± 6.3	39.6 ± 4.3^*^
*RI*_@50 kHz_	55.4 ± 6.8	39.5 ± 4.6^*^	57.7 ± 6.4	40.0 ± 4.4^*^
*ZI*_@250 kHz_	61.4 ± 7.2	43.6 ± 5.1^*^	64.0 ± 7.0	43.6 ± 4.7^*^
*RI*_@250 kHz_	61.3 ± 7.5	43.4 ± 5.1^*^	63.9 ± 7.1	43.*8* ± 4.8^*^
*ZI*_@500 kHz_	63.5 ± 7.5	45.0 ± 5.2^*^	66.2 ± 7.2	45.0 ± 4.8^*^
*ZI*_@1 MHz_	65.4 ± 7.7	46.3 ± 5.4^*^	68.1 ± 7.4	46.3 ± 5.0^*^
*ZI*_@2 MHz_	67.3 ± 7.9	47.6 ± 5.6^*^	70.1 ± 7.7	47.7 ± 5.1^*^
*ZI*_@3 MHz_	68.7 ± 8.1	48.6 ± 5.7^*^	71.6 ± 7.9	48.7 ± 5.2^*^

*BMI* Body bass index, *FFM* Fat-free mass, *FM* Body fat mass, *PBF* Percent body fat; *Xc*_@xkHz_: reactance at 5 kHz, 50 kHz and at 50 kHz; *R*_@xkHz_: resistance at 5 kHz, 50 kHz, and 250 kHz; *ZI*_@xkHz_: impedance index at 1 kHz, 5 kHz, 50 kHz, 250 kHz, 500 kHz, 1 MHz, 2 MHz and 3 MHz; *RI*_@xkHz_: resistance index at 50 kHz, 250 kHz; * = significantly different from women at *p* < 0.05; † = significantly differenct from development group at *p* < 0.05

There were no differences in age and BMI among all the groups. Men were higher and had more weight, FFM, ALM, and *Xc* at 250 kHz, and more *ZI* or *RI* at all frequencies than women in both groups (all *P* < 0.05). Men had less FM, and less *R* at 5 kHz, 50 kHz, and 250 kHz than women in both groups (all *P* < 0.05). *Xc* at 5 kHz, and *Xc* at 50 kHz were in no difference between men and women. The *Xc* at 5 kHz of women was higher than that of women in the cross-validation group (*P* < 0.05).

### Bivariate regression analyses of ALM as dependent and ZIs as independent

**Fig. 1:**
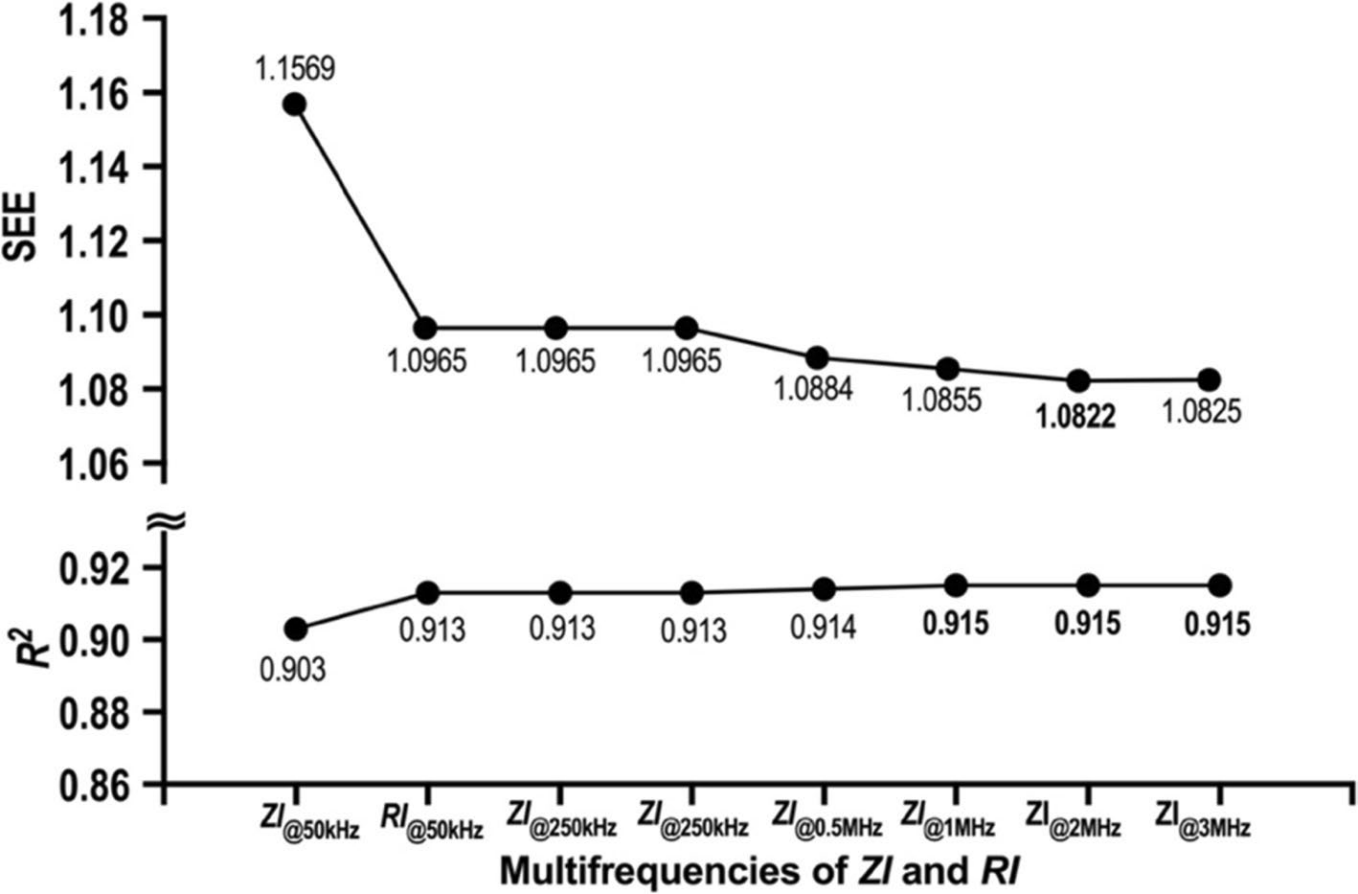
Predictive accuracy and coefficient of determination in each frequency of ZI and RI from the bivariate linear regression analysis

The results of SEE and *R*^2^ from each bivariate regression analysis inputting the impedance index of a specific frequency as an independent variable and as the dependent variable of ALM are shown in Fig. 1. Among the range of 0.5 to 3 MHz, *ZI* at 1 MHz, *ZI* at 2 MHz and *ZI* at 3 MHz showed the highest explanatory values of determinant coefficient, and among them, *ZI* at 2 MHz had the highest prediction accuracy according to the SEE.

### Development and Validation of MF-BIA Equations for ALM

**Fig. 2:**
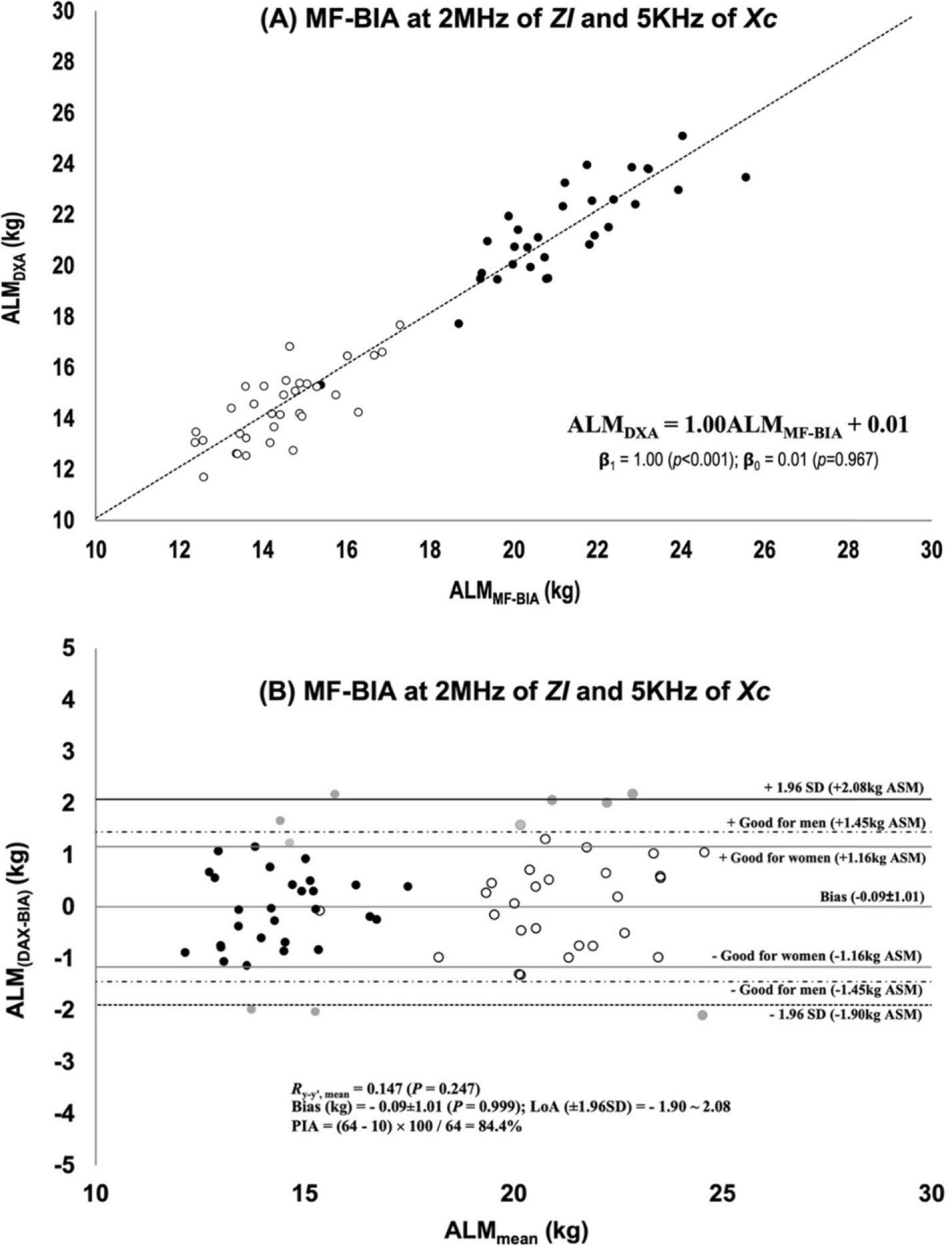
Bivariate regression analyses and Bland–Altman plot for MF-BIA at 2 MHz of ZI and 5 kHz of Xc (**A**) The line of best fit (**B**) Bland–Altman plot of the difference between ALM measured by DXA and predicted by MF-BIA from the cross-validation group; Ry-y’, mean = correlation coefficient between the bias and the mean of the reference and predicted ALM; = men and = women who are within the subjective rating for good, = men and women out of the subjective rating for good

For the validation study, the stepwise multiple regression analysis was used to develop the ALM equations on 2/3 of the total sample with the remaining 1/3 used for internal cross-validation as shown in Table 2. The regression models for the development sample for older people were used to predict ALM in the validation sample (Table 2). The predicted ALM in the cross-validation group (17.68 ± 3.75 kg) did not differ from the measured ASM in this group (17.77 ± 3.90 kg, *P* = 0.486) and the total error was 1.00 kg ALM in muscle-specific frequency at 2 MHz for *ZI*, subjectively rated as ““Excellent” in men and “Very Good” in women by Jeon’s [26] and Lohman’s criteria [8, 25, 27], which were similar to the SEEs in the development group. As shown in Fig. 2(A) for the line of best fit, the predicted ASM in the cross-validation group had a significant relation with the measured ALM with a slop of 1.00 (*P* < 0.001). The y-intercept showed no significant difference from zero (*P* = 0.967) by the bivariate linear regression analysis. In Fig. 2(B) for the Bland–Altman plots, the residual (ALM_(DXA-BIA)_) was not significantly correlated with ALM_mean_ (*R*_y-y’,mean_ = 0.147). LoA (± 1.96 SD) were between -1.90 and 2.08 kg ALM with the PAI as 81.8% at the 2 MHz muscle-specific frequency in the cross-validation group.

**Table 2:** Prediction equations for ALM developed on 2/3 of the sample and internal cross-validated on the remaining 1/3^a^

Validation for BIA-based *ALM* prediction equation
***Development group (n* = *131)***
Measured ALM = 17.28 ± 3.62
Predicted ALM = 0.254*ZI*_@2 MHz_ + 1.0324sex_M1F0_ + 0.066*Xc*_@5 kHz_ + 1.396
R = 0.964, Adusted R^2^ = 0.928, SEE = 0.97 kg, CV = 5.6%, SR: Excellent (M), Very good (F),
VIF: *ZI*_@2 MHz_ = 3.39, SEX_M1F0_ = 3.46, *Xc*_@5 kHz_ = 1.11
Predicted ASM = 17.25 ± 3.48
***Internal Cross-validation group (n* = *64)***
Measured ALM = 17.77 ± 3.90
Predicted ALM = 17.68 ± 3.75, *p* = 0.486^*^
* R* = 0.965, *R*^2^ = 0.931, TE = 1.00 kg, CV = 5.6%, SR: Excellent (M), Very Good (F), PIA: 84.4%

*ZI*_@2 MHz_ = impedance index at 2 MHz; SEX_M1F0_: man = 1, women = 0; *XC*_*@*5 kHz_ = reactance at 5 kHz; SEE (Standard Error of Estimate) =$\sqrt{\sum {(Measured ALM-Estimated ALM)}^{2}/(N-p-1)}$where *p* = number of predicter variables); SR = subject rating [ideal = 0.72 ~ 0.90(M), 0.54 ~ 0.65(F); excellent = 0.90 ~ 1.09(M), 0.65 ~ 0.83(F); very good = 1.09 ~ 1.27(M), 0.83 ~ 1.01(F); good = 1.27 ~ 1.45(M), 1.01 ~ 1.16(F); fairly good; fair; poor] [26]; TE = $\sqrt{\sum {(Measured ALM-Estimated ALM)}^{2}/N}$; PIA = the number of individuals within ± 1.45 kg ALM for men and ± 1.16 kg ALM for women of difference between predicted and measured ALM × 100 / total), * = paired t-test betwee measured ALM and predicted ALM

### The Final Muscle-Specific MF-BIA Prediction Equations for ALM

Thus, final equations using all the 195 older people were developed for appendicular muscle-specific multifrequency BIA prediction of ALM (Table 3). The *ZI*_@2 MHz_, the muscle-specific predictive factor, explained 91.5% of variability in ALM and accumulated *R*^2^ (combined with weight and a*Xc*_@5 kHz_) explained variability up to 93.0% by adjusted *R*^2^. The group predictive accuracy of SEE with subjective rating as “excellent” in men and “very good” in women for appendicular lean-specific MF-BIA prediction equation. For VIF, the newly developed equations showed no multicollinearity among variables. There was no significant mean difference in ALM between DXA and MF-BIA (17.44 ± 3.71 vs. 17.45 ± 3.58, *P* = 0.874).

**Table 3:** The final predictive BIA equation for ALM on Korean older people

Final Predictive Equation (*n* = 195)
Measured ALM = 17.44 ± 3.71
Predicted ALM = 0.247*ZI*_@2 MHz_ + 1.254sex_M1F0_ + 0.067*Xc*_@5 kHz_ + 1.739
*R* = 0.965, *Adusted R*^2^ = 0.930, SEE = 0.97 kg, SR: Excellent (M), Very good (F)
CV = 5.6%, VIF: *ZI*_@2 MHz_ = 3.54, SEX_M1F0 =_ 3.57, *Xc*_@5 kHz_ = 1.07
Predicted ALM = 17.45 ± 3.58

* = paired t-test betwee measured ALM and predicted ALM

### External Cross-Validation of Published Equations for ALM

Table 4 shows the results in the external cross-validation of the six published BIA equations for ALM comparing with ALM by DXA. In the equation array, there a moderate-to-high determinant coefficient between each ALM_BIA_ and ALM_DXA_ (*R*^2^ = 0.836 ~ 0.931, *P* < 0.001) expect for ALM_BIA_ by Scaroflieri, et al. The TEs of all published equations exceeded the acceptable subjective range as “poor”. The published equations by Vermeiren, et al., and Peniche, et al. showed 28.2% and 55.4% of PIA among equations that had a moderate determinant coefficient. The PIA of the equation by Vermeiren, et al. and the equation by Peniche, et al. were 28.2% and 55.4%, respectively, and the remaining published equations showed 0 or 0.05% of PIA. The new MF-BIA equation performed best in estimating the ALM with much higher *R*^2^ and more accurate TE into the acceptable subject rating as “excellent” for men and “very good” for women. In the Bland–Altman plot in the new one, there was no significant bias (-0.01 ± 0.97 kg, *P* = 0.875) and significant correlation coefficient in *R*_y-y’,mean_ between measured ALM and predicted ALM with 81.4% of PIA.

**Table 4:** External cross-validation of published bioimpedance-based equations for the prediction of ALM in 195 Asian Korean people in the current study

	Predicted ALM (kg)	*R*^2a^	Bias^b^ (kg)	LoA (kg)	*P*^c^	TE (kg)	Subjective Rating		*R*_y-y’, mean_^d^	PIA (%)
Measured ALM	17.44 ± 3.71						Male	Female		
***Supine position***										
Kim_@2MHz_BWA2.0_	17.45 ± 3.58	0.931	-0.01 ± 0.97	-1.92, 1.90	.875	0.97	Excellent	Very good	0.131 (*P* = .068)	82.1
Vermeiren, et al. [15]	15.66 ± 3.37	0.918	1.78 ± 1.08	-0.33, 3.89	.000	2.08	Poor	Poor	0.319 (*P* = .000)	32.8
Scafoglieri, et al. [17]	17.51 ± 1.86	0.630	-0.07 ± 2.50	-4.98, 4.84	.702	2.50	Poor	Poor	0.776 (*P* = .000)	28.2
Sergi, et al. [16]	23.62 ± 3.42	0.836	-6.18 ± 1.50	-9.12, -3.23	.000	6.36	Poor	Poor	0.199 (*P* = .005)	00.5
Kyle, et al. [19]	22.65 ± 4.09	0.847	-5.21 ± 1.60	-8.36, -2.07	.000	5.45	Poor	Poor	-0.245 (*P* = .000)	00.5
Kim, et al. [20]	9.41 ± 2.37	0.885	8.03 ± 1.68	4.74, 11.32	.000	8.21	Poor	Poor	0.806 (*P* = .000)	00.0
Peniche, et al. [18]	16.57 ± 3.99	0.845	0.87 ± 1.57	-2.21, 3.94	.000	1.79	Fair	Poor	-0.185 (*P* = .010)	55.4

^a^*R*^2^ = the coefficient of determination shared by measured and predicted ALM, ^b^Predicted minus measured value, ^c^*P*-value for paired t-test that bias = 0, ^d^*R*_y-y’, mean_ = correlation coefficient between the bias and the mean of the reference and predicted ALM

### The Overall Agreement of Diagnostic Classifications

The cut-offs for sarcopenia by AWGS [10] classified the values of ASMI obtained from the two BIA equations and the newly developed MF-BIA equation as shown Table 5. The overall agreement of the newly developed one was significantly rated as “substantial” (*k* = 0.779, *P* < 0.001), whereas that of the published BIA equations were significantly rated as “fair” (*k* = 0.216 ~ 3.00, *P* < 0.001). The new MF-BIA prediction equation had a specificity, PPV, and NPV of 90% or more with a sensitivity as 71.4%. The two published BIA equations were not able to determine sensitivity, specificity, PPV, and NPV because the level of overall agreement was unsatisfied.

**Table 5:** Prevalence, sensitivity and specificity of the acceptable BIA equations to determine sarcopenia

Equations	Overall Agreement *N* (%)	Cohen’s Kappa	Sensitivity	Specificity	PPV	NPV
BIA_@2 MHz_	185(94.9)	0.779*	71.4	98.8	91.3	95.3
BIA_Vermeiren_	108(55.4)	0.216*	100.0	47.3	25.6	100.0
BIA_Peniche_	143(73.3)	0.300*	70.0	73.9	32.8	93.1

AWGS Cut-off of ASMI: Female < 5.4 kg·m^−2^, Male < 7.0 kg·m^−2^, Agreement is poor if *k* < 0.00, slight if 0.00 < *k* < 0.20, fair if 0.21 < *k* < 0.40, moderate if 0.41 < *k* < 0.60, substantial if 0.61 < *k* < 0.80, and almost perfect if *k* > 0.80; * *P* < 0.001

## Discussion

The need for the impedance measures with a skeletal tissue-specific high-frequency that can be directly comparable to ALM measured by DXA in epidemiological and clinical settings has led us to develop and cross-validate accurate MF-BIA prediction equations for the ALM in elderly men and women. The MF-BIA prediction equation developed in this study included the impedance index (*ZI*) for the skeletal muscle-specific frequency at 2 MHz, reactance (*Xc*) at 5 kHz, and sex as variables. The accuracy and precision of the newly developed prediction equation for MF-BIA were high, not only at the group level (*R*^2^ = 0.931, SEE = 0.97 kg ALM) but also at the individual level (Bias = -0.01 ± 0.97, LoA = -1.92 ~ 1.90 kg ALM, PIA = 82.1%). The equation’s sensitivity, specificity, and overall agreement in the diagnosis of sarcopenia make its use suitable for clinical settings and epidemiological studies. Thus, it is demonstrated that the newly established appendicular lean-specific 2 MHz high-frequency BIA prediction equation can be applied to clinical settings and epidemiological studies with high accuracy.

The main achievement of the present study was the development of a new and accurate prediction equation that validates its within-group and individual error based on the 2 MHz high-frequency impedance index. Said index expresses the characteristics of appendicular lean mass, without anthropometric variables in the equation. Previous BIA prediction equations for appendicular lean mass included age, weight, and waist circumference as explanatory variables [14–20, 22, 25, 28], in addition to *ZI*, *RI*, and *Xc* as original variables of bioimpedance parameters to reduce the error from the assumption that the human body has a cylindrical conductive volume [29]. However, the use of multiple empirical variables (or anthropometric variables) in the regression, causes problems of multicollinearity linked to the unreliability of the estimated regression coefficient because of the correlation between explanatory variables and increased variance of the regression coefficient. This results in the derivation of an inaccurate regression equation [26, 30, 31].

This study excluded the explanatory variable of sex included in the prediction equation and added reactance based on the 2 kHz high-frequency impedance index, thereby ensuring that the VIF (*ZI* = 3.54, *Xc* = 1.07, *Sex* = 3.57) of each explanatory variable does not have multicollinearity. Therefore, each explanatory variable had a valid prediction equation with independent explanations for the ALM. In particular, the explanatory variability of the 2 MHz high-frequency impedance index had among the three independent variables an of *R*^2^ = 0.915 (*R* = 0.957) with SEE = 1.0822 kg AMS.

The explanatory variability and SEE of *ZI* or *RI* in the 50 kHz BIA prediction equations of previous studies were each: below 0.860 and above 1.34 kg ALM [28], 0.836 and above 1.450 kg ASM [15], below 0.852 and above 1.283 kg ALM [17], 0.883 and 1.401 kg ALM [16], 0.856 and 1.26 kg ALM [18]; and 0.917 and 1.53 kg ALM [19]. The explanatory variability and error of recently reported 250 kHz BIA prediction equations were each: 0.906 and 1.490 kg ALM [26], 0.415 ~ 0.635 and 2.27 ~ 1.88 kg ALM [14], and 0.825 and 1.35 kg ALM [20]. The addition of *Xc* of 5 kHz and sex along with *ZI* as predictor variables improved the explanatory variability and SEE of the prediction equation. Thus, the explanatory variance of *R*^2^ = 0.930 (*R* = 0.965) and group predictive accuracy of SEE = 0.97 in the new high-frequency prediction equation was superior to that of most previously published predictive equations (*R*^2^ = 75.7 ~ 92.5%, SEE = 1.02 kg ~ 1.46 kg) [15, 18, 19, 22, 26]. Its values were further comparable to results from the equations [16, 20] developed through large-scale studies by Peniche and Kyle which had predictive powers of *R*^2^ = 0.910 *R*^2^ = 0.952 and SEE = 1.01 kg and SEE = 1.12 kg ALM, respectively. Therefore, the appendicular lean-specific 2 MHz high-frequency impedance index used in this study showed the highest explanatory variability and precision for the single impedance index among all reported studies.

The reason for this is that the 2 MHz *ZI* has high explanatory variability specific to appendicular lean mass and induces conductivity ideal for the intracellular fluid, which composes up to 80% of skeletal muscles [11, 32]. To date, there have been reports of prediction equations for fat-free mass and appendicular lean mass using impedance index at 50 kHz, which is shown to have the highest reactance on the Cole–Cole model [13]. However, Deurenberg & Schouten [1992] reported a frequency range up to 1350 kHz that did not have high reactance, but high conductivity in both the extracellular and intracellular fluid through penetration of the cellular membrane [12]. Their study demonstrated that conductivity in both the extracellular and intracellular fluids under high frequency results in measurements of extracellular and intracellular fluids with higher precision and accuracy. However, the lack of advancements in technology and screening required for multifrequency measurements has led the 50 kHz index to be used most often hitherto. Said index also allows for simple measurements and conductivity of not only the extracellular but also the intracellular fluid [13, 33].

On the other hand, recent innovations in technology allowed high-multifrequency range measurements at 5 kHz, 50 kHz, 250 kHz, 500 kHz, 1 MHz, 2 MHz, and 3 MHz to be made easily and safely at lower frequencies, resulting in a higher accuracy of the measurements of the intracellular fluid. This would have enabled this study to precisely measure the conductivity of the intracellular fluid of skeletal muscle, which constitutes the highest proportion of total body water. Therefore, it may be predicted that high frequency ranges could be used in the conversion of *ZI* using the conductive volume model for appendicular skeletal muscles to ALM to obtain precise and accurate prediction equations in the future.

Simultaneously, this study had no bias in its individual predictive accuracy, and the percentage of individual agreement (PIA) within the margin of error was above 81%. In particular, this study had improved LoA of -1.90 ~  + 2.08 kg ALM in comparison to LoAs of previous studies such as: -2.68 ~ 2.41 kg ALM [28], -2.82 ~ 2.81 kg ALM [15], -2.467 ~ 2.562 kg ALM [17], -2.22 ~ 2.22 kg ALM [16], -2.23 ~ 2.46 kg ALM [18], -2.2 ~ 2.1 kg ALM [19], -1.95 ~ 1.98 kg ALM [26], and -2.2 ~ 1.9 kg ALM [20]. This improved LoA make the novel equation suitable for predicting individual ASMs.

The present study conducted an external cross-validation between previously existing prediction equations for the ASM of the older Korean population and those developed in this study. Acceptance standards for the predictive accuracy of regression equations were *R*^2^ above 0.800 without bias, SEE below 1.45 kg ALM for males, SEE below 1.16 kg ALM for females, and PIA above 70% [13, 26, 27, 30].

The two-MHz high-frequency BIA regression equation, the regression equation reported by Vermerien et al., and that reported by Perniche met the acceptance standards for group and individual precision. On the other hand, regression equations by Scafogliery, Sergi, Kyle, and Kim met standards for explanatory variance, but had large SEE, and had PIAs largely below the acceptance standard. This prevented these equations from meeting the acceptance standards for predictive accuracy and was unsuitable for application to older Korean people. The predictive equations reported by Scafogliery, Sergi, and Kyle were mostly developed based on Caucasian and African American populations, which made their application to Koreans presumably difficult. White and African Americans have a different relationship between muscle mass and body electrical conductivity and resistance due to their relatively shorter torso and longer limbs in comparison to East Asians and a higher body density in comparison to Asians [8, 13].

These discrepancies remark the need for the development of prediction equations specific to Asian populations. In contrast, while the BIA_Kim_ [20] developed based on Korean or Asian Seniors had the advantage of using a relatively high frequency of 250 kHz, the study shared the problems of low accuracy observed during development of the prediction equation and large margins of error to have the lowest accuracy overall. Indeed, the TE of BIA_Kim_ was 8.21 kg ALM in contrast to the TE of 0.97 kg ALM of the BIA prediction equation developed in this study [20]. This made it necessary for Kim’s prediction equation for Koreans and Asian populations to be replaced by the new prediction equation proposed in this study. The overall results of External Cross-Validation demonstrated that the prediction equation of Vermeiren, that by Peniche, and the prediction equation newly developed in this study were able to provide accurate predictions for Korean seniors in both group and individual levels.

Finally, BIA_@2 MHz_, BIA_Vermeiren_, and BIA_Peniche_ were verified to check their applicability for sarcopenia diagnosis. The two BIA prediction equations by Vermeiren and Peniche were 55.4% and 73.3% overall agreement and Cohen’s Kappa of 0.216 and 0.300, causing detailed analysis on sensitivity, specificity, positive predictive value, and negative predictive value to be impossible. In contrast, the newly developed appendicular lean-specific high-frequency BIA prediction equation had an overall agreement of 94.9% and Cohen’s Kappa of 0.779 (almost perfect), making evaluation possible due to its sensitivity, specificity, positive predictive value, and negative predictive value to be determined suitable for individual sarcopenia diagnoses and clinical application. In particular, the specificity, negative predictive value, and positive predictive value showed potential for accurate diagnosis above 90%, while the sensitivity of 71.4% was much higher than the previously reported 37%–55% [34] and showed improved results with a sensitivity of 63.3% from the recently reported multifrequency prediction equation using 250 kHz *ZI* and 50 kHz *Xc*. The improved values of sensitivity, specificity, positive predictive value, and negative predictive value would be applicable to epidemiological studies and clinical settings to a level comparable to that of DXA, unlike previous studies. As stated previously, such improvements would be based on the electrophysiological principle behind the usage of ZI within the 2 MHz–3 MHz range that allows sufficient conductivity in skeletal muscles [26, 32, 35]. It is recommended that future studies develop BIA prediction equations for appendicular skeletal muscle using ZI within the high frequency range of 2 MHz–3 MHz.

This study developed a new appendicular lean-specific high-frequency BIA prediction equation for the prediction of ALM based on 195 healthy older people aged 70 years and over in Korea. The relatively narrow range of age and BMI, living environment, the health status of the participants, and the sample size of the population under study, could affect the generalizability of our results. This study used DXA as a reference method for the ASM measurement. There are several limitations to using DXA to measure the ALM. ALM measurements were obtained by calculating the total muscle mass by subtracting the mineral content from the lean arm and leg mass. DXA does not separate skeletal muscle from the skin, connective tissue, or blood vessels [36, 37]. Thus, DXA may overestimate ALM [31, 32] compared to the gold standard (i.e., CT or MRI) to quantify body composition. Although there is a high correlation between DXA and the gold standard, DXA has been considered as the reference standard for the BIA model [3, 10, 38–40], fat infiltration that occurs in aging upon the replacement of water and connective tissue within muscle tissue with fat can have some influence on the accuracy of skeletal muscle mass measured using DXA [36, 41].

This study only investigated the elderly in the Seoul area of South Korea, so caution is required when generalizing its results to other populations. In addition, this study only measured the ALM of the elderly to diagnose sarcopenia, which has certain limitations in assessing sarcopenia in the elderly. Therefore, future research could add tests to diagnose severe sarcopenia (including muscle strength and performance), and also expand to different groups (children, adolescents and adults) and increase the sample size. Regarding the level of physical activity (PA), we recruited elcerly people who were apparently healthy and able to do activityes of daily living as the research participants. Participants' PA levels may also have an impact on BIA results. Future research should take morbidity/co-morbidities into consideration.

## Conclusion

In conclusion, the newly developed appendicular lean-specific high-frequency BIA prediction equation has a high predictive accuracy, sensitivity, specificity, and agreement for both individual and group measurements. Thus, the high-frequency BIA prediction equation is suitable not only for epidemiological studies, but also for the diagnosis of sarcopenia in clinical settings.

## Data Availability

The datasets generated during and/or analyzed during the study will be available from the corresponding author on reasonable request.

## Abbreviations

ALM: Appendicular lean muscle
ASMI: Appendicular skeletal muscle mass index
AWGS: Asian Working Group for Sarcopenia
BIA: Bioelectrical impedance analysis
BMI: Body mass index
ECF: Extracellular fluid
EWGSOP2: The European Working Group on Sarcopenia in Older People 2
FM: Fat mass
FFM: Fat-free mass
LoA: Limits of agreement
MF-BIA: Multifrequency bioelectrical impedance analysis
NPV: Negative prediction value
PIA: Percentage of individual agreement
PPV: Positive prediction value
*R*: Resistance
*RI*: Resistance index
SEE: Standard error of estimated
TBW: Total body water
WHO: The World Health Organization
*Xc*: Reactance
*Z*: Impedance
*ZI*: Impedance index

## References

[1] Rosenberg IH (2011). Sarcopenia: origins and clinical relevance. Clin Geriatr Med.

[2] Anker SD, Morley JE, von Haehling S (2016). Welcome to the ICD-10 code for sarcopenia. J Cachexia Sarcopenia Muscle.

[3] Cruz-Jentoft AJ, Bahat G, Bauer J, Boirie Y, Bruyere O, Cederholm T (2019). Sarcopenia: revised European consensus on definition and diagnosis. Age Ageing.

[4] Studenski SA, Peters KW, Alley DE, Cawthon PM, McLean RR, Harris TB (2014). The FNIH sarcopenia project: rationale, study description, conference recommendations, and final estimates. J Gerontol A Biol Sci Med Sci.

[5] Chen LK, Lee WJ, Peng LN, Liu LK, Arai H, Akishita M (2016). Recent Advances in Sarcopenia Research in Asia: 2016 Update From the Asian Working Group for Sarcopenia. J Am Med Dir Assoc.

[6] Fielding RA, Vellas B, Evans WJ, Bhasin S, Morley JE, Newman AB (2011). Sarcopenia: an undiagnosed condition in older adults. Current consensus definition: prevalence, etiology, and consequences. International working group on sarcopenia. J Am Med Dir Assoc..

[7] Cruz-Jentoft AJ, Baeyens JP, Bauer JM, Boirie Y, Cederholm T, Landi F (2010). Sarcopenia: European consensus on definition and diagnosis: Report of the European Working Group on Sarcopenia in Older People. Age Ageing.

[8] Buckinx F, Landi F, Cesari M, Fielding RA, Visser M, Engelke K (2018). Pitfalls in the measurement of muscle mass: a need for a reference standard. J Cachexia Sarcopenia Muscle.

[9] Heymsfield SB, Gonzalez MC, Lu J, Jia G, Zheng J (2015). Skeletal muscle mass and quality: evolution of modern measurement concepts in the context of sarcopenia. Proc Nutr Soc.

[10] Chen LK, Woo J, Assantachai P, Auyeung TW, Chou MY, Iijima K, et al. Asian Working Group for Sarcopenia: 2019 Consensus Update on Sarcopenia Diagnosis and Treatment. J Am Med Dir Assoc. 2020;21:300-7e2. 10.1016/j.jamda.2019.12.012 32033882

[11] Lukaski HC (1996). Biological indexes considered in the derivation of the bioelectrical impedance analysis. Am J Clin Nutr.

[12] Deurenberg P, Schouten FJ (1992). Loss of total body water and extracellular water assessed by multifrequency impedance. Our J Clan Nutr..

[13] Gonzalez MC, Barbosa-Silva TG, Heymsfield SB (2018). Bioelectrical impedance analysis in the assessment of sarcopenia. Curr Opin Clin Nutr Metab Care.

[14] Yamada Y, Nishizawa M, Uchiyama T, Kasahara Y, Shindo M, Miyachi M (2017). Developing and Validating an Age-Independent Equation Using Multi-Frequency Bioelectrical Impedance Analysis for Estimation of Appendicular Skeletal Muscle Mass and Establishing a Cutoff for Sarcopenia. Int J Environ Res Public Health.

[15] Vermeiren S, Beckwee D, Vella-Azzopardi R, Beyer I, Knoop V, Jansen B (2019). Evaluation of appendicular lean mass using bio impedance in persons aged 80+: A new equation based on the BUTTERFLY-study. Clin Nutr.

[16] Sergi G, De Rui M, Veronese N, Bolzetta F, Berton L, Carraro S (2015). Assessing appendicular skeletal muscle mass with bioelectrical impedance analysis in free-living Caucasian older adults. Clin Nutr.

[17] Scafoglieri A, Clarys JP, Bauer JM, Verlaan S, Van Malderen L, Vantieghem S (2017). Predicting appendicular lean and fat mass with bioelectrical impedance analysis in older adults with physical function decline - The PROVIDE study. Clin Nutr.

[18] Rangel Peniche DB, Raya Giorguli G, Alemán-Mateo H (2015). Accuracy of a predictive bioelectrical impedance analysis equation for estimating appendicular skeletal muscle mass in a non-Caucasian sample of older people. Arch Gerontol Geriatr.

[19] Kyle UG, Genton L, Hans D, Pichard C (2003). Validation of a bioelectrical impedance analysis equation to predict appendicular skeletal muscle mass (ASMM). Clin Nutr.

[20] Kim JH, Choi SH, Lim S, Kim KW, Lim JY, Cho NH (2014). Assessment of appendicular skeletal muscle mass by bioimpedance in older community-dwelling Korean adults. Arch Gerontol Geriatr.

[21] Janssen I, Heymsfield SB, Baumgartner RN, Ross R (1985). Estimation of skeletal muscle mass by bioelectrical impedance analysis. J Appl Physiol.

[22] Bosy-Westphal A, Jensen B, Braun W, Pourhassan M, Gallagher D, Müller MJ (2017). Quantification of whole-body and segmental skeletal muscle mass using phase-sensitive 8-electrode medical bioelectrical impedance devices. Eur J Clin Nutr.

[23] Visser M, Deurenberg P, van Staveren WA (1995). Multi-frequency bioelectrical impedance for assessing total body water and extracellular water in elderly subjects. Eur J Clin Nutr.

[24] Foster KR, Lukaski HC (1996). Whole-body impedance–what does it measure?. Am J Clin Nutr.

[25] Kim Chul-Hyun (2020). Validation of an 50kHz sigle frequncy BIA equation for appendicular skeletal muscle mass of old people comparable to DXA. Korean J Meas Eval Physical Educ Sport Sci.

[26] Jeon KC, Kim SY, Jiang FL, Chung S, Ambegaonkar JP, Park JH (2020). Prediction Equations of the Multifrequency Standing and Supine Bioimpedance for Appendicular Skeletal Muscle Mass in Korean Older People. Int J Environ Res Public Health.

[27] Anand G, Yu Y, Lowe A, Kalra A. Bioimpedance analysis as a tool for hemodynamic monitoring: overview, methods and challenges. Physiol Meas. 2021;42:10.1088/1361-6579/abe80e. 10.1088/1361-6579/abe80e 33607637

[28] Toselli S, Campa F, Matias CN, de Alencar Silva BS, Dos Santos VR, Maietta Latessa P, et al. Predictive equation for assessing appendicular lean soft tissue mass using bioelectric impedance analysis in older adults: Effect of body fat distribution. Exp Gerontol. 2021;150:111393. 10.1016/j.exger.2021.111393 33965554

[29] Organ LW, Bradham GB, Gore DT, Lozier SL (1985). Segmental bioelectrical impedance analysis: theory and application of a new technique. J Appl Physiol.

[30] Heyward VH, Wagner DR (2004). Applied body composition assessment.

[31] Kim JH (2019). Multicollinearity and misleading statistical results. Korean J Anesthesiol.

[32] Faes TJ, van der Meij HA, de Munck JC, Heethaar RM (1999). The electric resistivity of human tissues (100 Hz-10 MHz): a meta-analysis of review studies. Physiol Meas.

[33] Kyle UG, Bosaeus I, De Lorenzo AD, Deurenberg P, Elia M, Manuel Gomez J (2004). Bioelectrical impedance analysis-part II: utilization in clinical practice. Clin Nutr.

[34] De Rui M, Veronese N, Bolzetta F, Berton L, Carraro S, Bano G (2017). Validation of bioelectrical impedance analysis for estimating limb lean mass in free-living Caucasian elderly people. Clin Nutr.

[35] Bartels EM, Sorensen ER, Harrison AP (2015). Multi-frequency bioimpedance in human muscle assessment. Physiol Rep..

[36] Erlandson MC, Lorbergs AL, Mathur S, Cheung AM (2016). Muscle analysis using pQCT. DXA and MRI Eur J Radiol.

[37] Clark RV, Walker AC, Miller RR, O’Connor-Semmes RL, Ravussin E, Cefalu WT. Creatine ( methyl-d3) dilution in urine for estimation of total body skeletal muscle mass: accuracy and variability vs. MRI and DXA J Appl Physiol. 1985;2018(124):1–9. 10.1152/japplphysiol.00455.2016 PMC6048459 28860169

[38] Tosato M, Marzetti E, Cesari M, Savera G, Miller RR, Bernabei R (2017). Measurement of muscle mass in sarcopenia: from imaging to biochemical markers. Aging Clin Exp Res.

[39] Fuller NJ, Hardingham CR, Graves M, Screaton N, Dixon AK, Ward LC (1999). Assessment of limb muscle and adipose tissue by dual-energy X-ray absorptiometry using magnetic resonance imaging for comparison. Int J Obes Relat Metab Disord.

[40] Koster A, Ding J, Stenholm S, Caserotti P, Houston DK, Nicklas BJ (2011). Does the amount of fat mass predict age-related loss of lean mass, muscle strength, and muscle quality in older adults?. J Gerontol A Biol Sci Med Sci.

[41] Segal KR, Burastero S, Chun A, Coronel P, Pierson RN, Wang J (1991). Estimation of extracellular and total body water by multiple-frequency bioelectrical-impedance measurement. Am J Clin Nutr.

