# Correction: Two-megahertz impedance index prediction equation for appendicular lean mass in Korean older people

**DOI:** 10.1186/s12877-022-03122-3

**Published:** 2022-06-28

**Authors:** Hyeoijin Kim, Keon-Hyoung Song, Jatin P. Ambegaonkar, Sochung Chung, Kwonchan Jeon, Fang Lin Jiang, Jin Jong Eom, Chul-Hyun Kim

**Affiliations:** 1 Department of Physical Education, Korean National University of Education, Cheongju, Republic of Korea; 2 Department of Pharmaceutical Engineering, Soonchunhyang University, Asan, Republic of Korea; 3 SMART Laboratory, School of Kinesiology, George Mason University, Manassas, VA USA; 4 Department of Pediatrics, Konkuk University Medical Center, Konkuk University School of Medicine, Seoul, Republic of Korea; 5 School of Health Sciences, Public Health Program, Salisbury University, Salisbury, MD USA; 6 Department of Sports Medicine, Soonchunhyang University, Asan, Republic of Korea; 7 Department of Sport, Leisure & Recreation, Soonchunhyang University, Asan, Republic of Korea

**Correction: BMC Geriatr 22, 385 (2022)**

**https://doi.org/10.1186/s12877-022-02997-6**

After publication of this article [1], the authors reported that in this article the author Fang Lin Jiang was incorrectly linked to affiliations 6 and 7, but should have been linked to affiliation 6 only; and the author Chul-Hyun Kim was incorrectly linked to affiliation 7, but should have been linked to affiliation 6.

The original article [1] has been updated.
